# Archaeal contribution to carbon-functional composition and abundance in China’s coastal wetlands: Not to be underestimated

**DOI:** 10.3389/fmicb.2022.1013408

**Published:** 2022-11-10

**Authors:** Meiling Yang, Na Liu, Baoli Wang, Yajun Li, Jianfeng Li, Cong-Qiang Liu

**Affiliations:** ^1^School of Earth System Science, Institute of Surface-Earth System Science, Tianjin University, Tianjin, China; ^2^Bohai Coastal Critical Zone National Observation and Research Station, Tianjin University, Tianjin, China

**Keywords:** bacteria and archaea, carbon-functional genes, community relationship, ecological driver, labile Fe

## Abstract

Microbial diversity, together with carbon function, plays a key role in driving the wetland carbon cycle; however, the composition, driving factors of carbon-functional genes and the relationship with microbial community have not been well characterized in coastal wetlands. To understand these concerns, microbes, carbon-functional genes, and related environmental factors were investigated in twenty wetlands along China’s coast. The results indicate that carbon-functional gene composition is dominated by archaeal rather than bacterial community and that Nanoarchaeaeota is the dominant archaeal phylum associated with carbon cycling in anoxic sediments. Compared with microbes, carbon-functional composition was more stable because they showed the highest Shannon diversity and archaeal functional redundancy. Deterministic processes dominated microbial community, and stochastic processes were more important for carbon-functional genes. Labile Fe governed archaeal and carbon-functional composition by coupling with nitrogen and carbon biogeochemical cycles, while bacterial community was affected by NH_4_-N and SOC/SON. This study highlights the predominant contributions of archaea to carbon-functional genes and to the stability of carbon-functional composition, thus providing new insights into the microbial dominance of the carbon cycle and the evaluation of carbon function in coastal wetlands.

## Introduction

Soil/sediment carbon (C) has a vital role in regulating climate, nutrient cycling and biodiversity and provides ecosystem services that are essential to human well-being (Victoria et al., 2012; LaRowe et al., 2020). Carbon processes (e.g., carbon fixation and mineralization) are mainly driven by microorganisms, which release enzymes encoded by corresponding carbon-functional genes (Chen and Sinsabaugh, 2021). A comprehensive investigation of the composition, diversity, and abundance of microbial taxonomy and carbon-functional genes is essential in understanding microbially mediated carbon biogeochemical processes.

Microbes, as hosts of functional genes and ecosystem components, have historically been seen as engines driving Earth’s biogeochemical cycles (Falkowski et al., 2008). Carbon-functional genes have been deemed direct participants in carbon cycling because of their strong relationships with the activities of their corresponding carbon-metabolic enzymes (Trivedi et al., 2016). However, the relationship between microbial community and microbial function is not well understood. Most previous studies focused on microbial taxa composition and functional prediction (Qiu et al., 2021), assembly mechanisms (Zhou and Ning, 2017), and the effects of microbial taxa diversity on ecosystem multifunctionality (Wagg et al., 2014; Delgado-Baquerizo et al., 2017). Only a few studies have highlighted that the prediction of element-cycling processes can be improved more by functional gene abundance than by relying on microbial diversity (Graham et al., 2016). The lack of knowledge about carbon-functional genes dominated by microbes creates an obstacle to directly predicting and assessing of the carbon cycling in ecosystem, even in targeted microbial culture and function studies. Therefore, establishing the links between microbial community and function and revealing the dominant microbes of functional genes are of great significance for understanding the carbon-cycling processes of ecosystems.

The functional genes carried by microbes theoretically vary with microbial composition and abundance, but functional redundancy usually makes functional composition more stable than does microbial community in the same habitat (Chen et al., 2022). Microbial community assembly was discerned by niche theory and neutral theory (Zhang et al., 2022), which have found that the relative importance of deterministic factors (abiotic and biotic factors) and stochastic processes (birth, death, speciation, limited dispersal and immigration) depends on regional and local environmental conditions (Zhou and Ning, 2017; Wang B. L. et al., 2021). To the best of our knowledge, the assembly process of carbon-functional structure is largely unknown, but a functional gene matrix can form a special microbiome involved in the element cycle and has been regarded as a community based on function rather than on taxonomic groups (Dong et al., 2021; Zhou et al., 2021). Microbes have great diversity and incredible numbers, and this once created significant challenges for establishing direct links between microbes and function and identifying the redundant complexity. However, with the development of high-throughput sequencing technology, we can get information about microbial composition and diversity with 16S rRNA sequencing (Mughini-Gras et al., 2021). Carbon-functional genes can be obtained with a high-throughput quantitative-PCR-based chip (HT-qPCR QMEC) (Zheng et al., 2018). Thus, taking carbon-functional genes as a special community, using the above techniques to comprehensively study the ecological drivers of carbon-functional genes and their predominant microbes will fill the gaps in the accurate evaluation and prediction of the predominantly microbial-driven carbon cycle.

Coastal wetlands have a high carbon sequestration capacity and a low decomposition rate. This sequestered carbon is often called “coastal blue carbon” together with carbon buried in seagrass bed ecosystem (Deb and Mandal, 2021; Wang F. M. et al., 2021). Microscopic bacteria and archaea play decisive roles in the sequestration of organic carbon and the transformation of organic carbon into inorganic carbon. There are many studies on wetland microbial communities and diversity, but information on carbon-functional genes in coastal wetlands is limited, especially across multiple types of wetlands and at large spatial scales. Therefore, we collected 36 sediment samples from 20 wetlands along the eastern coast of China in order to evaluate and quantify carbon-functional genes and analyze microbial communities. We aimed to address a set of fundamental questions to improve our understanding of the dominant microbes driving the coastal wetland carbon cycle: (i) What taxonomic groups and carbon-cycling functional traits are present in coastal wetland sediments? (ii) Which microbes dominate the composition and abundance of carbon-functional genes? (iii) What are the ecological drivers of microbial community and carbon-function composition? (iv) What environmental factors affect microbial community and carbon-functional composition?

## Materials and methods

### Study area and sampling

**FIGURE 1:**
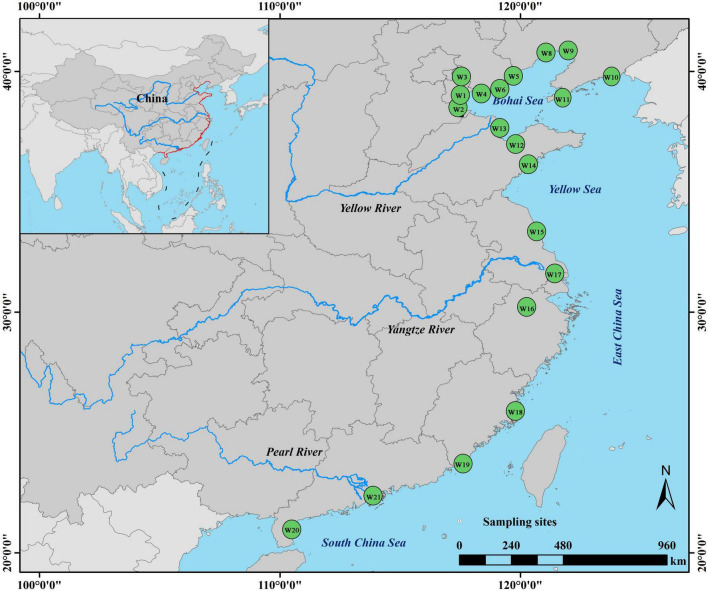
Geographic map of sampling sites in China’s coastal wetlands.

China’s coastal wetlands are characterized by tropical, subtropical, and temperate monsoon climates, and their temperature differences in summer are small. The main vegetation in coastal wetlands is comprised of *Phragmites communis*, *Acorus calamus*, and mangroves. Twenty coastal wetlands (from W1 to W21, except for W7) in eastern China were selected for this study (Figure 1); their types are diverse and include four reservoirs, one lagoon, four estuaries, four rivers, two marshes, one bay, one delta, and three mangrove wetlands (Supplementary Table 1). In total, 36 sediment samples were collected from July to August, 2019 (Supplementary Table 1). Samples were collected from vegetated and open areas in the central location of the wetland using a KH0202 layered box corer (Kanghua Inc., China). Three to five sediment columns were obtained from each of the two sampling areas, and then the surface sediments (depth: 1–5 cm) were mixed in 50 mL sterilized centrifuge tubes. All samples were refrigerated in dry ice and directly transported to the laboratory. Sediment samples for microbial taxonomic and carbon-functional analysis were stored at −80°C prior to DNA extraction, and those for physicochemical property analysis were stored at −20°C. In accordance with sediment sampling, overlying water was collected and immediately used for physicochemical analysis.

### Physicochemical analysis

Overlying water temperature (WT), chlorophyll a (Chl a), dissolved oxygen (DO), salinity (Sal), and pH were measured directly using a Professional Plus multi-parameter probe (YSI, EXO1, America) with precorrection *in situ*. Dissolved organic carbon (DOC) was determined by a TOC analyzer (Aurora 1030, America). Comprehensive trophic level index (TLI) was calculated on a weighted basis for TN, TP, and Chl a (Lu et al., 2017). Physicochemical properties of sediment pore water, including labile PO_4_-P, NO_3_-N, NH_4_-N (measured by ZrO-AT DGT) and Fe (measured by ZrO-Chelex DGT), were analyzed by Diffusive Gradients in Thin-films (DGTs) (Ren et al., 2020). Sediment samples were ground and sieved through a 2 mm nylon sieve after lyophilization at −50°C for further analysis. Sediment organic carbon (SOC), sediment organic nitrogen (SON), and SOC/SON ratio were measured with an elemental analyzer (Vario EL III, Germany). Values for δ^13^C_SOC_ and δ^15^N_SON_ were determined by MAT253 Plus-EA (Thermo Fisher, America). Dissolved organic matter (DOM) composition was analyzed by Fourier transform ion cyclotron resonance mass spectrometry (FT-ICR MS, Bruker solariX 2XR, Germany) coupled with electrospray ionization (ESI) (Brünjes et al., 2022). Sediment particle mean diameter was determined using a Malvern laser grain-size analyzer (Mastersizer 3000, England) after sieving and removing organic matter and carbonates (Yu et al., 2018). Sediment moisture content (MC) was calculated by gravimetric method as MC = (W_wet_−W_dry_)/W_wet_.

### Amplicon sequencing and carbon-functional gene analysis

Genomic DNA was extracted from 0.3 g sediment samples with the TGuide S96 Magnetic Soil DNA Kit (Tiangen Biotech Co., Ltd., Beijing, China). The quality and integrity of extracted DNA was assessed with the Thermo Scientific Microplate Reader (Multiskan GO, America). The primer sets targeting 16S rRNA genes of microbes (Zhang and Lu, 2016; Chen et al., 2021) and functional genes associated with microbial carbon metabolism (Zheng et al., 2018) were selected for PCR amplification as described in Supplementary Tables 2, 3. 16S rRNA sequencing was performed on the Illumina NovaSeq 6000 platform, and HT-qPCR QMEC was used for genetic quantification (Zheng et al., 2018). Both of projects were carried at Meige Technology Co., Ltd., Guangzhou, China. Bioinformatic analysis of amplicon sequencing and detailed protocols of HT-qPCR QMEC were performed as described in S1. The sequences reported in this study have been deposited in NCBI SRA database with the BioProject numbers PRJNA681135 and PRJNA674461 (Wang B. L. et al., 2021). Shannon index and niche breadth of microbes and carbon-functional genes were conducted with the “vegan_2.6-2” and “spaa_0.2.2” package, respectively.

### Data analysis

iCAMP, a phylogenetic bin-based null model was applied to understand assembly processes that determined the formation of microbial community and potential community functionalities (Ning et al., 2020). Microbial OTUs and functional genes were first assigned to phylogenetic-closed groups (bins); and then bin-based null model simulations with phylogenetic diversity was used for partitioning homogeneous and heterogeneous selection of deterministic processes, whereas taxonomic diversity was used for partitioning dispersal limitation, homogenizing dispersal, and undominated (drift, diversification, weak selection, and/or weak dispersal) in stochastic processes (Ning et al., 2020). The proportion of the ecological process in different communities can be compared based on the bootstrapped methods, and the analyses were conducted with the “iCAMP v.1.3.4” packages in R 3.6.3.

Correlation-based network analysis was used for microbes and carbon-functional gene interactions (Zhu et al., 2017; Wang B. L. et al., 2021). Co-occurrence networks were constructed using WGCNA libraries in R 3.6.3 and visualized with Gephi 0.9.2 software. OTUs present in ≥10% of all samples and all carbon-functional genes were retained for analysis. Pairwise Spearman’s correlations were calculated between OTUs, with a correlation coefficient of >0.7 and a *P*-value < 0.05 (Benjamini and Hochberg adjusted) being considered to indicate a valid relationship.

Random Forest model analysis was conducted to identify dominant microbes of carbon-functional genes using the “Random Forest” package (Edwards et al., 2018). Mantel test was used for correlational analysis between microbial community composition and carbon-functional genes according to the *r* and significance-level *P*-values of the two matrices. Spearman’s correlation analyses between physicochemical factors and microbial community structure and carbon-functional genes were calculated using the core function in the corrplot_0.92 package. The significant differences between different groups were analyzed by one-way analysis of variance (ANOVA) and least significant difference (LSD) at the 0.05 significance level using the SPSS 24.0 statistical software package.

## Results

### Physicochemical parameters

The mean diameter of sediment particles ranged from 7.61 to 179.43 μm, with an average of 50.06 μm, and moisture content was in the range of 18.35∼78.4%, with an average of 47.85% (Table 1). Except for a few sampling points, there was less variability in concentrations of DGT-PO_4_-P, DGT-NH_4_-N, and DGT-Fe from south to north, while DGT-NO_3_-N concentration varied greatly within a range of 0.06∼0.41 mg.L^–1^ (Supplementary Figure 1). The contents of SOC and SON changed little overall, but their ratios varied across a wide range (Supplementary Figure 1). The average values of δ^13^C_SOC_ and δ^15^N_SON_ were −26.263 and 5.346‰ (Table 1). Values for TLI, Chl a, pH, DO, and DOC had disorderly spatial variations. Water temperature generally decreased from south to north, while salinity increased (Supplementary Figure 1). DOM molecules were classified into eight classes of chemical components based on the different O/C and H/C ratios (Supplementary Table 4). Aliphatic/protein was the most dominant chemical component, accounting for 47.0%, followed by 28% lignin/CRAM-like structures and 14% lipids. The DOM Shannon index ranged from 0.89 to 1.51 (Table 1).

**TABLE 1:** Physicochemical parameters and DOM α-diversity index of surface sediments and overlying water in China’s coastal wetlands.

Parameters	Units	Min	Max	Ave	SD	*n*
Latitude	°	21.11	40.95			36
MD	μm	7.61	179.43	50.06	40.08	36
MC	%	18.35	78.04	47.85	12.80	33
DOM Shannon		0.89	1.51	1.29	0.15	36
DGT-PO_4_-P	mg.L^–1^	0.00	0.45	0.07	0.12	33
DGT-NO_3_-N	mg.L^–1^	0.06	0.41	0.14	0.08	33
DGT-NH_4_-N	mg.L^–1^	0.00	0.46	0.03	0.08	32
DGT-Fe	mg.L^–1^	0.02	5.50	0.66	1.32	33
SON	g.kg^–1^	0.14	37.53	5.51	8.58	35
SOC	g.kg^–1^	0.69	338.22	44.28	65.87	36
SOC/SON		4.88	14.32	9.18	2.36	35
δ^13^C_SOC_	‰	–30.786	–20.449	–26.263	2.471	36
δ^15^N_SON_	‰	–0.287	13.379	5.346	3.364	36
TLI		43.79	79.41	62.19	9.78	32
Chl a	μg L^–1^	3.65	229.36	69.66	76.76	34
WT	°C	21.0	36.0	29.0	3.0	34
pH		6.79	9.26	8.03	0.60	34
DO	mg L^–1^	0.01	19.57	6.84	4.31	34
Sal	‰	0	30.6	5.80	7.46	34
DOC	mg L^–1^	0.87	46.88	8.80	10.01	35

Min, the minimum; Max, the maximum; Ave, the average; SD, standard deviation; *n*, the number of measurements. Sediment-related index: sediment particle mean diameter (MD); sediment moisture content (MC); α-diversity index of dissolved organic matter (DOM Shannon); sediment organic nitrogen (SON); sediment organic carbon (SOC); ratio of SOC to SON (SOC/SON); overlying water-related index: comprehensive trophic level index (TLI); chlorophyll a (Chl a); water temperature (WT); dissolved oxygen (DO); salinity (Sal); dissolved organic carbon (DOC).

### Composition of microbial communities and carbon-functional genes

**FIGURE 2:**
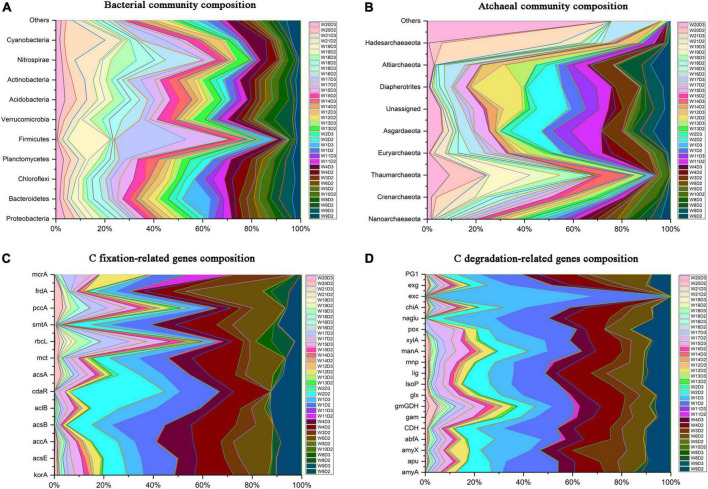
Composition and spatial distribution of microbial community and carbon-functional genes. Top bacterial phyla **(A)**; all archaeal phyla **(B)**; carbon fixation-related genes **(C)**; carbon degradation-related genes **(D)**.

A total of 53, 503 OTUs in bacteria and 21, 350 OTUs in archaea were obtained. All bacterial OTUs were clustered into 73 phyla, and the most frequently detected phyla were Proteobacteria (13∼62%), Bacteroidetes (7∼63%), Chloroflexi (0.5∼17%). For archaea, Nanoarchaeaota (1.8∼87%), Crenarchaeota (4.2∼86%), and Thaumarchaeaota (0.3∼83%) were the dominant phyla (Figures 2A,B). In total, 13 carbon fixation-related genes (*accA*, *aclB*, *acsA*, *acsB*, *acsE*, *frdA*, *cdaR*, *korA*, *mcrA*, *mct*, *pccA*, *rbcL*, and *smtA*) and 19 carbon degradation-related genes (amyA, *amyX*, *apu*, *gam*, *IsoP*, *gmGDH* and *exg* for starch; *manA*, *xylA* and *abfA* for hemicellulose; *CDH* and *naglu* for cellulose; *chiA*, and *exc* for chitin; *glx*, *lig*, *mnp* and *pox* for lignin; and *PG1* for pectin) were obtained (Figures 2C,D). The bacterial relative abundance was generally homogeneous from low latitude to high latitude, and that of archaea (e g., Crenarchaeota and Thaumarchaecta) decreased from low latitude to high latitude (Figures 2A,B), while the abundance of carbon-functional genes was lower in low latitude (Figures 2C,D).

### α-diversity and niche breadth of microbial communities and carbon-functional genes

**FIGURE 3:**
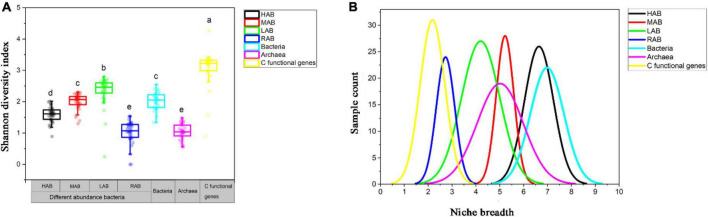
Shannon diversity index **(A)** and niche breadth **(B)** of bacteria, archaea, and carbon-functional genes. The relevant abbreviations are referred to the text. Significant difference between groups are indicated with lowercase letters (*p* < 0.05).

All the bacteria were classified into four groups: high abundance (HAB), with OTUs of a relative abundance > 1% in all samples; medium abundance (MAB), with OTUs of a relative abundance 0.1∼0.9% in all samples; low abundance (LAB), with OTUs of a relative abundance 0.01%∼0.09% in all samples; and rare abundance (RAB), with OTUs of a relative abundance 0.001%∼0.009% (Nyirabuhoro et al., 2020). The highest Shannon diversity index was found in carbon-functional genes with an average of 3.07, and was lowest in archaea with an average of 1.05 (Figure 3A). For bacteria, the Shannon diversity index was highest for LAB at 2.34, and the lowest value for bacteria was 1.60 (Figure 3A). In contrast, the niche breadth of carbon-functional genes was the smallest, with an average of 2.18 (Figure 3B); the niche breadth of archaea was smaller than that of bacteria; and bacterial niche breadth decreased with decreasing abundance (Figure 3B).

### The relationships between microbial communities and carbon-functional genes

**FIGURE 4:**
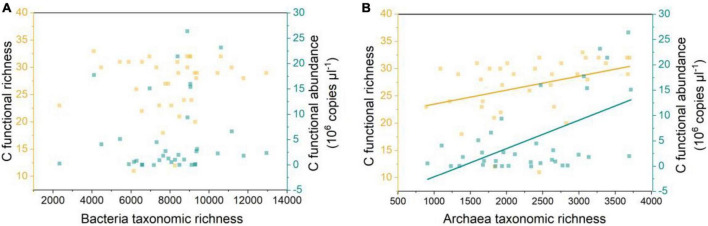
Bacteria taxonomic richness vs. carbon-functional richness and carbon-functional gene absolute abundance **(A)**; archaea taxonomic richness vs. carbon-functional richness and carbon-functional gene absolute abundance **(B)**; the regression (yellow): *y* = 0.003 x + 20.91, adjust *r*^2^ = 0.11, *p* < 0.05; the regression (green): *y* = 5.61 × 10^3^ × –7.64 × 10^6^, adjust *r*^2^ = 0.31, *p* < 0.05.

**FIGURE 5:**
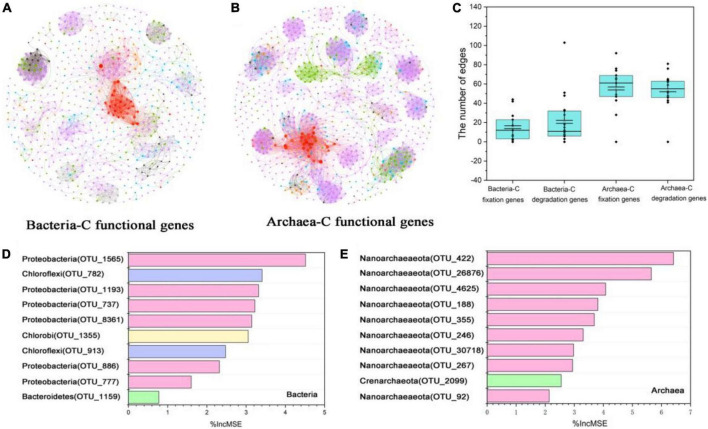
Co-occurrence networks and Random Forest analysis of microbes and carbon-functional genes. Co-occurrence networks of bacteria and carbon-functional genes **(A)**; co-occurrence networks of archaea and carbon-functional genes **(B)**; the numbers of edges of microbes (bacteria and archaea) and carbon-functional genes (fixation and degradation genes) based on co-occurrence networks **(C)**; Random Forest analysis of bacterial contribution to absolute abundance of carbon-functional genes **(D)**; Random Forest analysis of archaea contribution to absolute abundance of carbon-functional genes **(E)**.

Archaeal taxonomic richness was positively correlated with carbon-functional richness and absolute abundance, however, bacteria had no such correlations (Figures 4A,B). Archaea and carbon-functional genes co-occurrence networks, with 862 nodes and 1, 736 edges, were more than the 679 nodes and 640 edges for bacteria; the average clustering coefficient between archaea and carbon-functional genes was 0.782, which was higher than the 0.709 between bacteria and genes (Figures 5A–C and Supplementary Table 5). Random Forest analysis found the variable explanation of bacteria and archaea for carbon-functional gene abundance to be 40 and 59.68%, respectively (Figures 5D,E). The maximum%IncMSE of OTU annotated to Proteobacteria bacteria was 4.5%, while that of OTU annotated to Nanoarchaeaeota archaea was 6.41% (Figures 5D,E).

### Ecological and environmental drivers of microbial communities and carbon-functional genes

**FIGURE 6:**
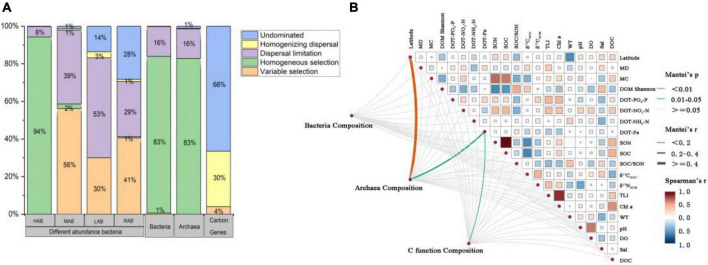
The assembly process of microbial communities and carbon-functional genes **(A)** and their relationship with environmental factors **(B)**. Pairwise comparisons of physicochemical factors are displayed with a color gradient to denote Spearman’s correlation coefficients. Community composition is related to each environmental factor by performing a Mantel test. The relevant abbreviations are referred to the text and Table 1.

The assembly processes of both bacterial and archaeal communities were dominated by homogeneous selection, and dispersal limitation only accounted for 16% (Figure 6A). Quite differently, carbon-functional communities were dominated by 66% undominated and 30% homogenizing dispersal processes, while variable selection only explained 4% (Figure 6A). HAB community was mainly dominated by homogeneous selection, while community composition for MAB, LAB, RAB were mainly dominated by variable selections and dispersal limitation (Figure 6A). Among all environmental variables, DGT-Fe was a main influencing factor for archaea and carbon-functional composition, and latitude also had an important effect on the archaea community (Figure 6B). The factors influencing bacterial community were more complex and different with abundance. DGT-NH_4_ was related to MAB community, while SOC/SON was related to RAB community (Supplementary Figure 2).

## Discussion

### Predominant archaea in sediments and their contributions to the composition and abundance of carbon-functional genes

As a significant fraction of Earth’s microbial diversity, archaea have been significant not only to the ecology of our planet but also to the evolution of eukaryotes (Castelle et al., 2015; Uri and Neta, 2022). Unlike bacteria, archaea live in extreme conditions, at low and high temperatures, extreme pH, elevated pressure and high salinity (Ciaramella et al., 2005), as well as in normal natural environments such as soil (Tripathi et al., 2015) and fresh water (Juottonen et al., 2020). As a special realm in aquatic ecosystem, coastal sediment is rich in organic matters and therefore hosts a large number of archaea (Wang et al., 2012; Flemming and Wuertz, 2019). We found higher archaea ratios in China’s coastal wetlands sediment than in water (Supplementary Figure 3) and a significant correlation between archaea taxonomy and carbon-functional genes (Figure 4 and Supplementary Figures 4, 5), confirming the predominant role of archaea in the carbon cycling of sediments.

Sediment is hypoxic and anaerobic but rich in organic matter, and this special environment may require more microbes with unique or complex carbon metabolic mechanisms in order to promote the transformation and circulation of various forms of carbon, while archaea with a narrower niche breadth than bacteria tend to be more specialized (Figure 3B). Archaea are involved in many metabolic processes, such as the Wood-Ljungdahl (WL) pathway (He et al., 2016; Adam et al., 2017), the reductive citric acid cycle (Hügler et al., 2003), and the 3-hydroxy propionate/4-hydroxybutyrate (3-HP/4-HB) and dicarboxylic acid/4-hydroxybutyric acid (Sato and Atomi, 2011) pathways, which differ from previously recognized classical pathways related to autotrophic carbon fixation. For example, the newly discovered methanogenic archaea (*Candidatus Methanoliparum*), independent on bacteria, can directly oxidize long-chain alkanes and enter methanogenic metabolism through β-oxidation and WL pathway (Zhou et al., 2022). Thaumarchaeota, the dominant group of chemoautotrophs, can use the chemical energy generated during nitrification to fix CO_2_ and synthesize organic compounds with the 3-HP/4-HB cycle in oligotrophic and deep lakes (Zhang et al., 2012; Könneke et al., 2014). Besides, most archaea have the potential to degrade or participate in the metabolism of complex macromolecular organic matter such as proteins and aromatic compounds (Li et al., 2015; Adam et al., 2017). Thus, archaea’s more diverse functions in carbon cycling determine its dominance in carbon-functional gene richness (Figure 4B). The discovery of more archaeal linkages with carbon-functional genes in co-occurrence networks reconfirms archaea’s dominant contribution to carbon-functional genes (Figures 5B,C). Nanoarchaeaeota have been determined by genome sequencing to drive key ecological processes such as glycolysis, fermentation and gluconeogenesis (Baker et al., 2020), and our results show that they play key roles in carbon cycle processes (Figure 5E and Supplementary Figure 5). Although archaea phyla have been found in much smaller numbers than bacteria phyla (Figure 2), and are the least-studied and least-characterized domain of life (Baker et al., 2020; Uri and Neta, 2022), the findings above remind us not to ignore or undervalue archaea, which may perform more-or more specific-functions than bacteria do in driving the carbon biogeochemical cycle in coastal wetland sediment.

### More direct indication of carbon-function genes for wetland ecosystem carbon function

The carbon-functional genes had higher evenness and stability and a slight distance decay (Figure 3 and Supplementary Figure 6). Functional redundancy should be a strong support for the stability of carbon function, and the altered functional structure should be alleviated by microbes performing similar or identical ecological functions under environmental disturbance (Louca et al., 2018). Archaea (the lowest slope of 0.398) and HAB (slop of 0.522) were highly functionally redundant compared with other bacteria (Supplementary Figure 7), and these individual microbes can avoid the effects of environmental disturbance on coordination or deterioration of microbes and the disappearance of individuals so as to ensure normal ecological functions. MAB, LAB, and RAB had narrower niche breadth and low functional redundancy (slopes of 0.672, 0.602, 0.575) (Figure 3B and Supplementary Figure 7), which may be because most of them are species with unique functions, and so it is difficult to resist environmental disturbances with functional and individual substitute. From this point of view, the microbiome is composed of high-abundance generalized species and non-high-abundance specialized species, which together guarantee the carbon biogeochemical cycle through abundance superiority and functional complementarity. Therefore, it is not accurate to evaluate a change in ecosystem function only by the change of microbial community, because community and function changes were not synergistic. Similarly, soil carbon processes were thought not to be affected by microbial community composition (Zheng et al., 2019). As a more direct indicator, a change in carbon-functional gene composition and abundance can better reflect a change in ecosystem carbon function.

### Influential environmental factors for microbial communities and carbon-functional genes

In nature, the transmission of microbes and genes is both passive and active, and the relative importance of the assembly process shaping microbial communities and carbon-functional genes should be the result of reciprocal selection. We found that microbes were more susceptible to selective processes than were genes (Figure 6A), because they are more sensitive to abiotic environmental disturbance and to biological interactions, while diversity in taxonomic composition, abundance, and activity determine selection intensity (Yang et al., 2020; Wang B. L. et al., 2021). On the contrary, the stability of DNA molecular structure and microbial functional redundancy make carbon-functional genes more stable than their host microbes, and small environmental fluctuations have little impact on gene composition, so the assembly of carbon-functional gene composition is less affected by the selective process (Figure 6A).

**FIGURE 7:**
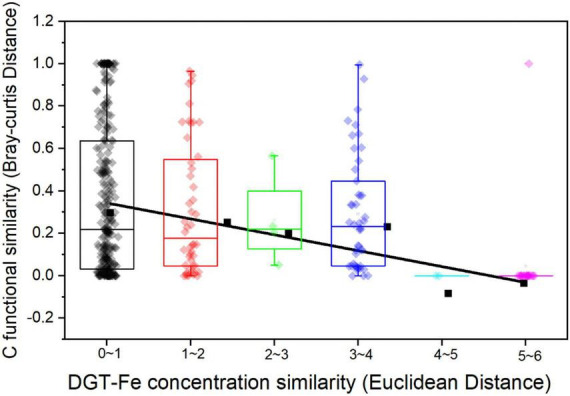
Relationships for DGT-Fe concentration similarity and carbon functional similarity. The regression: *y* = –0.06, *x* = +0.40, *r*^2^ = 0.65, *p* < 0.05.

DGT-Fe governs archaeal and carbon-functional composition (Figure 6B), which should be related to the key ecological functions of archaea. As a limiting factor, iron not only has its metal attributes, but also drives the element biogeochemical cycle by coupling with other elements (Conway and John, 2014). Ammonium-oxidizing archaea and methanogens were found to be involved in anaerobic ammonium oxidation coupling to Fe(III) reduction (Feammox) with dinitrogen gas, nitrite, and nitrate as the terminal products in the oligotrophic environment (Zhu et al., 2022). In coastal sediments, the negative correlation of DGT-Fe with NO_3_^–^ and NH_4_^+^ reinforced the importance of iron in the wetland nitrogen cycle driven by archaea (Figure 6B). Moreover, the abundance of genes associated with iron and carbon cycling often has a consistent change tendency in a given environment (Yang et al., 2017), and a previous study has verified that the microbial metabolic process of Fe(III) reduction is coupled with the mineralization of SOC as a terminal electron acceptor or with the formation of a complex with SOC (Chen et al., 2020). In this study, we also found DGT-Fe to have strong correlations with SOC/SON and carbon-functional gene composition (Figures 6B, 7), which should be that iron ultimately changed the SOC/SON by influencing the composition of carbon-functional genes and the intensity of carbon cycling.

Microbial communities regulate ecosystem fluxes of carbon, nitrogen with mineralization, and immobilization, and also have the ability to adapt to differences in resources composition and can thereby adjust their element use efficiencies (Ren et al., 2016). But because of the diversity of species composition and specificity to the environment, different microbial communities are affected by different environmental factors. The significant relationships of MAB communities with DGT-NH_4_-N reflect their ability to utilize nitrogen (Supplementary Figure 2), while those of RAB with SOC/SON suggested that they may be strictly homeostatic with constrained C/N and more sensitive to the imbalance of SOC/SON (Supplementary Figure 2).

## Conclusion

This work found that archaea dominate the richness and abundance of carbon-functional genes, and that Nanoarchaeaeota contribute the most abundance. Carbon-functional composition is more stable with the highest Shannon diversity because of microbial functional redundancy. DGT-Fe was the only factor found to affect the carbon-functional composition by coupling with nitrogen and carbon biogeochemical cycles. This study reinforces the view that there are significant differences in assembly process and environmental influence factors of microbial communities and carbon-functional genes. It is necessary to consider further the composition and abundance of carbon-functional genes rather than simply microbial community when assessing the carbon cycle intensity of coastal wetlands, because carbon-functional genes are more direct carbon participant encoding carbon-metabolism enzymes.

## Data availability statement

The datasets presented in this study can be found in online repositories. The names of the repository/repositories and accession number(s) can be found below: https://www.ncbi.nlm.nih.gov/, PRJNA681135 and https://www.ncbi.nlm.nih.gov/, PRJNA674461.

## Author contributions

BW, MY, and C-QL conceived the ideas and designed the methodology. MY, NL, and YL collected the data. MY, NL, YL, and JL analyzed the data. BW and MY led the writing of the manuscript. All authors contributed critically to the drafts and gave final approval for publication.
