# Supplementary material for: Archaeal contribution to carbon-functional composition and abundance in China’s coastal wetlands: Not to be underestimated
Source: Front Microbiol. 2022 Nov 10;13:1013408. doi: 10.3389/fmicb.2022.1013408 (PMC9685420; doi:10.3389/fmicb.2022.1013408)
Supplement: Supplementary file 1 [file Data_Sheet_1.docx]

**Archaeal Contribution to Carbon-Functional Composition and Abundance in China’s Coastal Wetlands: not to be Underestimated**

**Meiling Yang^1^, Na Liu^1^, Baoli Wang^1, 2*^, Yajun Li^1^, Jianfeng Li^1^, Cong-Qiang Liu^1, 2^**

^1^ Institute of Surface-Earth System Science, School of Earth System Science, Tianjin University, Tianjin, China

^2^ Bohai Coastal Critical Zone National Observation and Research Station, Tianjin University, Tianjin, China

*** Correspondence:**Baoli Wang
[baoli.wang@tju.edu.cn](mailto:baoli.wang@tju.edu.cn)

S1. Methods and Material

S1.1 Bioinformatic Analysis of 16S rRNA Sequencing

The obtained 16S rRNA raw reads were quality-filtered as previously described (Wang et al., 2021). The overlapping paired-end reads with sequence mismatching < 5 bp and alignment similarity > 90% were merged as clean tags using FLASH after quality filtration (Magoc and Salzberg, 2011). The clean tags with similarity at 97% consistency level were regarded as an Operational Taxonomic Units (OTUs) using UPARSE (Edgar, 2013). The sequences were chimeras detected using UCHIME (Edgar et al., 2011). After removal of the chimeras and singleton OTUs representing sequencing errors, the representative OTUs with the highest occurrence frequencies were assigned using the RDP, SILVA, and GREENGENES databases to get taxonomy annotation (Lan et al., 2012; Quast et al, 2013; DeSantis et al; 2006). Each sample was rarefied to the minimum sequencing depth (the minimum number of bacterial and archaeal reads is 97715 and 76346, respectively) to equalize sequencing depth (goods_coverage > 0.96), and sequence normalization was performed using MOTHUR v.1.33.3 (Schloss et al., 2009).

**S.1.2 HT-qPCR QMEC**

Amplification was conducted in a 100 nL reaction system, with all qPCR reactions conducted in triplicate for each primer set, and a non-template (sterile water) negative control was included for each run. Amplifying and melting processes were automatically generated and analyzed by the WaferGen software. The detection of each gene in each sample and its threshold cycle (Ct)_value was performed with Canoco software at Meige Technology Co., Ltd., Guangzhou, China. Multiple_melting peaks and peaks with amplification efficiencies less than 1.8 or greater than 2.2 were discarded. A threshold cycle of 31 was used as the detection limit. 16S rRNA was used as reference gene to standardize the data and obtain the relative quantification of each gene in each sample (Zhu et al., 2017). The absolute quantitative information of genes was obtained based on the absolute abundance of the 16S rRNA gene (Zhu et al., 2017).

**References**

DeSantis, T.Z., Hugenholtz, P., Larsen, N., Rojas, M., Brodie, E.L., Keller, K., Huber, T., Dalevi, D., Hu, P., Andersen, G.L., 2006. Greengenes, a chimera-checked 16S rRNA gene database and workbench compatible with ARB. Appl Environ Microb. 72(7):5069-5072. https://doi.org/10.1128/AEM.03006-05

Edgar, R.C., 2013. UPARSE: Highly accurate OTU sequences from microbial amplicon reads. Nat Methods. 10,996-998. <https://doi.org/10.1038/NMETH.2604>

Edgar, R.C., Haas, B.J., Clemente, J.C., Quince, C., Knight, R., 2011. UCHIME improves sensitivity and speed of chimera detection. Bioinformatics. 27(16),2194-2200. https ://doi.org/10.1093/bioinformatics/btr381

Lan, Y.M., Wang, Q., Cole, J.R., Rosen, G.L., 2012. Using the RDP classifier to predict taxonomic novelty and reduce the search space for finding novel organisms. Plos One. 7(3), e32491. <https://doi.org/10.1371/journal.pone.0032491>

Quast, C., Pruesse, E., Yilmaz, P., Gerken, J., Schweer, T., Yarza, P., Peplies, J., Glöckner, F.O., 2013. The SILVA ribosomal RNA gene database project: improved data processing and web-based tools. Nucl Acids Res. 41(D1):D590-D596. <https://doi.org/10.1093/nar/gks1219>

Schloss, P.D., Westcott, S.L., Ryabin, T., Hall, J.R., Hartmann, M., Hollister, E.B., Lesniewski, R.A., Oakley, B.B., Parks, D.H., Robinson, C.J., Sahl, J.W., Stres, B., Thallinger, G.G., Van Horn, D. J., Weber, C. F., 2009. Introducing mothur: open‑source, platform‑independent, community supported software for describing and comparing microbial communities. Appl Environ Microb. 75,7537. <https://doi.org/10.1128/AEM.01541-09>

Magoc, T., Salzberg, S.L., 2011. FLASH: fast length adjustment of short reads to improve genome assemblies. Bioinformatics. 27:2957-2963. https://doi.org/10.1093/bioinformatics/btr507

Wang, B.L., Liu, N., Yang, M.L., Wang, L.J., Liang, X., Liu C.Q., 2021. Co-occurrence of planktonic bacteria and archaea affects their biogeographic patterns in China’s coastal wetlands. Environ Microbiome. 16,19. <https://doi.org/10.1186/s40793-021-00388-9>

Zhu, Y.G., Zhao, Y., Li, B., Huang, C.L., Zhang, S.Y., Yu, S., Chen, S.Y., Zhang, T., Gillings, M.R., Su, J.Q., 2017. Continental-scale pollution of estuaries with antibiotic resistance genes. Nat Microbiol. 2,16270. https://doi.org/10.1038/nmicrobiol.2016.27

Table S1 Sampling information of twenty China’s coastal wetlands

| **Coastal wetland** | **Sampling sites** | **Sampling time** | **Logitude** | **Latitude** | **Location** | **Type** | **Vegetation** |
| --- | --- | --- | --- | --- | --- | --- | --- |
| Beidagang | W1D2; W1D3 | 2019.07.02 | 117°23'55" | 38°46'2" | Tianjin, China | Reservoir | *Phragmites communis* |
| Nandagang | W2D2; W2D3 | 2019.07.02 | 117°29'28" | 38°32'33" | Cangzhou, Hebei province, China | Reservoir | *Phragmites communis* |
| Qilihai | W3D2 | 2019.07.03 | 117°28'58" | 39°17'2" | Tianjin, China | Reservoir | *Phragmites communis* |
| Caofeidian | W4D2; W4D3 | 2019.07.04 | 118°22'19" | 39°11'9" | Tangshan, Hebei province, China | Reservoir | *Phragmites communis* |
| Qilihai | W5D2 | 2019.07.07 | 119°16'5" | 39°34'46" | Qinhuangdao, Hebei province, China | Lagoon | *Phragmites communis* |
| Luan River Estuary | W6D2 | 2019.07.07 | 119°15'32" | 39°25'8" | Qinhuangdao, Hebei province, China | Estuary | *Phragmites communis* |
| Suizhou | W8D2; W8D3 | 2019.07.09 | 120°21'14" | 40°20'46" | Huludao, Liaoning province, China | River | *Phragmites communis*  *Nymphaea tetragona* |
| Liao River Estuary | W9D2; W9D3 | 2019.07.10 | 121°46'38" | 40°56'46" | Panjin, Liaoning province, China | Estuary | *Phragmites communis* |
| Yalvjiang | W10D2 | 2019.07.12 | 123°38'5" | 39°55'26" | Dandong, Liaoning province, China | River | *Phragmites communis* |
| Qianguan | W11D2; W11D3 | 2019.07.13 | 121°39'45" | 39°2'7" | Dalian, Liaoning province, China | Marsh | *Phragmites communis* |
| Hetao | W12D2; W12D3 | 2019.07.18 | 119°54'56" | 37°12'10" | Laizhou, Shandong province, China | River | *Acorus calamus* |
| Yellow River Estuary | W13D2; W13D3 | 2019.07.19 | 119°1'25" | 37°45'46" | Dongying, Shandong province, China | Estuary | *Phragmites communis* |
| Shaohai | W14D2; W14D3 | 2019.07.21 | 120°6'4" | 36°15'17" | Jiaozhou, Shandong province, China | River | *Phragmites communis* |
| Dandinghe | W15D2; W15D3 | 2019.07.22 | 120°31'35" | 33°36'28" | Yancheng, Jiangsu province, China | Bay | *Acorus calamus* |
| Xixi | W16D2; W16D3 | 2019.07.24 | 120°4'25" | 30°16'15" | Hangzhou, Zhejiang province, China | Marsh | *Lemna minor*  *Eichhornia crassipes* |
| Changjiang Delta | W17D2; W17D3 | 2019.08.15 | 121°13'56" | 31°44'1" | Shanghai, China | Delta | *Phragmites communis*  *Acorus calamus* |
| Minjiang Estuary  Mangrove | W18D2; W18D3 | 2019.08.17 | 119°37'41" | 26°1'26" | Fuding, Fujian, China | Mangrove; Estuary | Mangrove |
| Zhangjiang Estuary Mangrove | W19D2; W19D3 | 2019.08.19 | 117°25'21" | 23°55'8" | Zhangzhou, Fujian, China | Mangrove; Estuary | Mangrove |
| Zhanjiang Mangrove | W20D2; W20D3 | 2019.08.21 | 110°18'54" | 21°6'22" | Zhanjiang, Guangdong, China | Mangrove | Mangrove |
| Nansha | W21D2; W21D3 | 2019.08.23 | 113°38'18" | 22°36'21" | Zhongshan, Guangdong, China | Estuary | *Phragmites communis* |

**Table S2** The primers of 16S rRNA sequencing

|  | **Amplified region** | **Primer name** | **Forward sequence** | **Reverse sequence** | **PCR type** |
| --- | --- | --- | --- | --- | --- |
| Bacteria | V4-1 | 515F/806R | GTGCCAGCMGCCGCGGTAA | GGACTACHVGGGTWTCTAAT | Conventional PCR |
| Archaea | V4-4 | 340F/1000R | CCCTAYGGGGYGCASCAG | GGCCATGCACYWCYTCTC | Nested PCR |
|  |  | 519F/806R | CAGYMGCCRCGGKAAHACC | GGACTACNSGGGTMTCTAAT |  |

**Table S3** The information of carbon-functional genes using HT-qPCR QMEC

| **No.** | **Gene_name** | **Classification** | **Encoded protein** | **Forward sequence** | **Reverse sequence** |
| --- | --- | --- | --- | --- | --- |
| 1 | *acc A* | C fixation | acetyl-CoA carboxylase carboxyltransferase α subunit | GAAGGCTAYCGCAARGC | CCTTCMGGSGARATMAC |
| 2 | *acl B* | C fixation | ATP-citrate lyase β subunit | TGGACMATGGTDGCYGGKGGT | ATAGTTKGGSCCACCTCTTC |
| 3 | *acs A* | C fixation | acetyl-coenzyme A synthetase | GATACCTGGTGGCAGACCGA | TGATCACGTCGTCGACCCGG |
| 4 | *acs B* | C fixation | acetyl-CoA synthase complex β subunit | CTYTGYCAGTCMTTYGCBCC | CCCATAAABCCYGGDGTYTG |
| 5 | *acs E* | C fixation | 5-methyltetrahydrofolate corrinoid methyltransferase | TCATCGGCGAACGCATCAAC | AGRCCGGCTTCSATGGC |
| 6 | *cda R* | C fixation | carbohydrate diacid regulon transcriptional regulator | CGARATGGTGGTGCTCAA | CARCGTRTTACGATGAATA |
| 7 | *frd A* | C fixation | fumarate reductase flavoprotein subunit | MTGCTGCACACSCTGTW | CCGGTSGGGTGRWACTG |
| 8 | *kor A* | C fixation | 2-oxoglutarate ferredoxin oxidoreductase α subunit | GCCGGCTACCCCATCACCCC | ATGATGGGATGGTCGCCATG |
| 9 | *mct* | C fixation | mesaconyl-CoA C1-C4 CoA transferase | TGGGCGCSGASGTSATMCG | TTGACSGTRTARTCSAYSGC |
| 10 | *mcr A* | C fixation | methyl-coenzyme M reductase α subunit | GGTGGTGTMGGDTTCACMCARTA | CGTTCATBGCGTAGTTVGGRTAGT |
| 11 | *pcc A* | C fixation | acetyl/propionyl-CoA carboxylase alpha | GTGMTGATCAAGGCCWC | CGSGTGTTCATYTCSAGGAA |
| 12 | *rbc L* | C fixation | ribulose-bisphosphate carboxylase large chain | AAGGAYGACGAGAACATC | TGCAGSATCATGTCRTT |
| 13 | *smt A* | C fixation | succinyl-CoA:(S)- malate CoA transferase | TTTCTGGCCGGBTAYGCDGC | CGGTACGGHCCGGTYTGVCC |
| 14 | *gam* | Starch hydrolysis | glucoamylase | CGSAACTGGGAYTACCGS | TCCCACAGSCCSKCGTC |
| 15 | *abf A* | Hemicellulose hydrolysis | α-L-arabinofuranosidase | CGSTAYCCSGGCGGCAAYTT | TGCCASGGNCCGTCCATYTC |
| 16 | *xylA* | Hemicellulose hydrolysis | xylose isomerase | TGGGGBGGTCGYGAAGG | ACTTTGGCRTCRAAGTT |
| 17 | *exg* | Cellulose hydrolysis | exoglucanase | YSTACGGSATGCACTGGMT | TANCGCAGRTAGTCVCCCAT |
| 18 | *PG1* | Pectin hydrolysis | pectinase/polygalacturonase | ANCATTGGTGGCCSTGGAA | TTRAYGGCRATRCARTCRTC |
| 19 | *mnp* | Lignin hydrolysis | manganese peroxidase | MACRCCSTTCGACTCSACC | ACGTCSGAGCAGTCRAYGA |
| 20 | *gmGDH* | Starch hydrolysis | glucose dehydrogenase | ATCGCGTTCGGGCCGGACG | ATSAGRTTSAGCTCGTCCCA |
| 21 | *IsoP* | Starch hydrolysis | Isopullulanase | GTCATYTACTTYGGNCC | CGNGCSACATCNGCCCA |
| 22 | *amyX* | Starch hydrolysis | pullulanase | TATAAYTGGGGMTATGAYCC | CCCATYAAATCAAAWCGRAA |
| 23 | *apu* | Starch hydrolysis | amylopullulanase | ACVTGGATAGGYGAGCCYCA | CCRTCSGGGAAGTAGTTKCC |
| 24 | *amyA* | Starch hydrolysis | α-amylase | YGGTTTTCGTCTTGACGCSG | MGGCTGMGTRTCATGRTTK |
| 25 | *manA* | Hemicellulose hydrolysis | β- mannanase | ATGCGCGGBGTCAACCA | TCGTTGSCGATGTTGABGA |
| 26 | *cdh* | Cellulose hydrolysis | cellobiose dehydrogenase | ATWRYCTWCCGMRTHGCCMT | GTKAGSGGRTTBYKGRYCAT |
| 27 | *naglu* | Cellulose hydrolysis | α-N-acetylglucosaminidase | TVAAYTGGTAYCTGAAATAY | CCRTGYAGVGCCATCCAGTC |
| 28 | *chiA* | Chitin hydrolysis | endochitinase | TSAAGAARTACGCSGACAACG | ASGTCATCAGRCCCTTSAG |
| 29 | *exc* | Chitin hydrolysis | exochitinase | GATTGGTSVCAATATGAYRG | STCCARCCACCRAYRCTRAA |
| 30 | *glx* | Lignin hydrolysis | glyoxal oxidase | AACCAGTCGATCATCTACGA | RTGSACGAGCTCDGGCATGG |
| 31 | *lig* | Lignin hydrolysis | lignin peroxidase | CCGCACACACTGTTGCTGC | CGAAGGATTGCCACTCGCA |
| 32 | *pox* | Lignin hydrolysis | phenol oxidase | ACYAGTATCCATTGGCACGGT | AGATGVGARTGATACCARAA |
| 33 | *16S rRNA* | 16S rRNA | ribosome | GGGTTGCGCTCGTTGC | ATGGYTGTCGTCAGCTCGTG |

**Table S4** Chemical components of surface sediment DOM in China**^’^**s coastal wetland

| **Parameters** | **Units** | **Min** | **Max** | **Ave** | **SD** | **n** |
| --- | --- | --- | --- | --- | --- | --- |
| Aliphatic/Proteins | % | 0.14 | 0.71 | 0.47 | 0.14 | 36 |
| Aromatic structures | % | 0.00 | 0.08 | 0.01 | 0.02 | 36 |
| Carbohydrates | % | 0.01 | 0.04 | 0.03 | 0.01 | 36 |
| Lignin/CRAM-like structures | % | 0.13 | 0.53 | 0.28 | 0.10 | 36 |
| Lipids | % | 0.04 | 0.34 | 0.14 | 0.07 | 36 |
| Tannins | % | 0.00 | 0.18 | 0.05 | 0.04 | 36 |
| Unsaturated hydrocarbons | % | 0.00 | 0.04 | 0.01 | 0.01 | 36 |
| Unclassified | % | 0.00 | 0.04 | 0.02 | 0.01 | 36 |

^a^Min, the minimum; Max, the maximum; Ave, the average; SD, standard deviation; n, the number of the measurement

**Table S5** Topological properties parameters of microbial communities and carbon-functional genes co-occurrence

| **Samples** | **Total nodes** | **Total edges** | **Average degree** | **Average clustering coefficient** | **Average path length** | **Modularity** | **Density** |
| --- | --- | --- | --- | --- | --- | --- | --- |
| Bacteria | 679 | 640 | 10.312 | 0.709 | 6.968 | 0.866 | 0.015 |
| Archaea | 862 | 1736 | 17.213 | 0.782 | 5.547 | 0.893 | 0.02 |


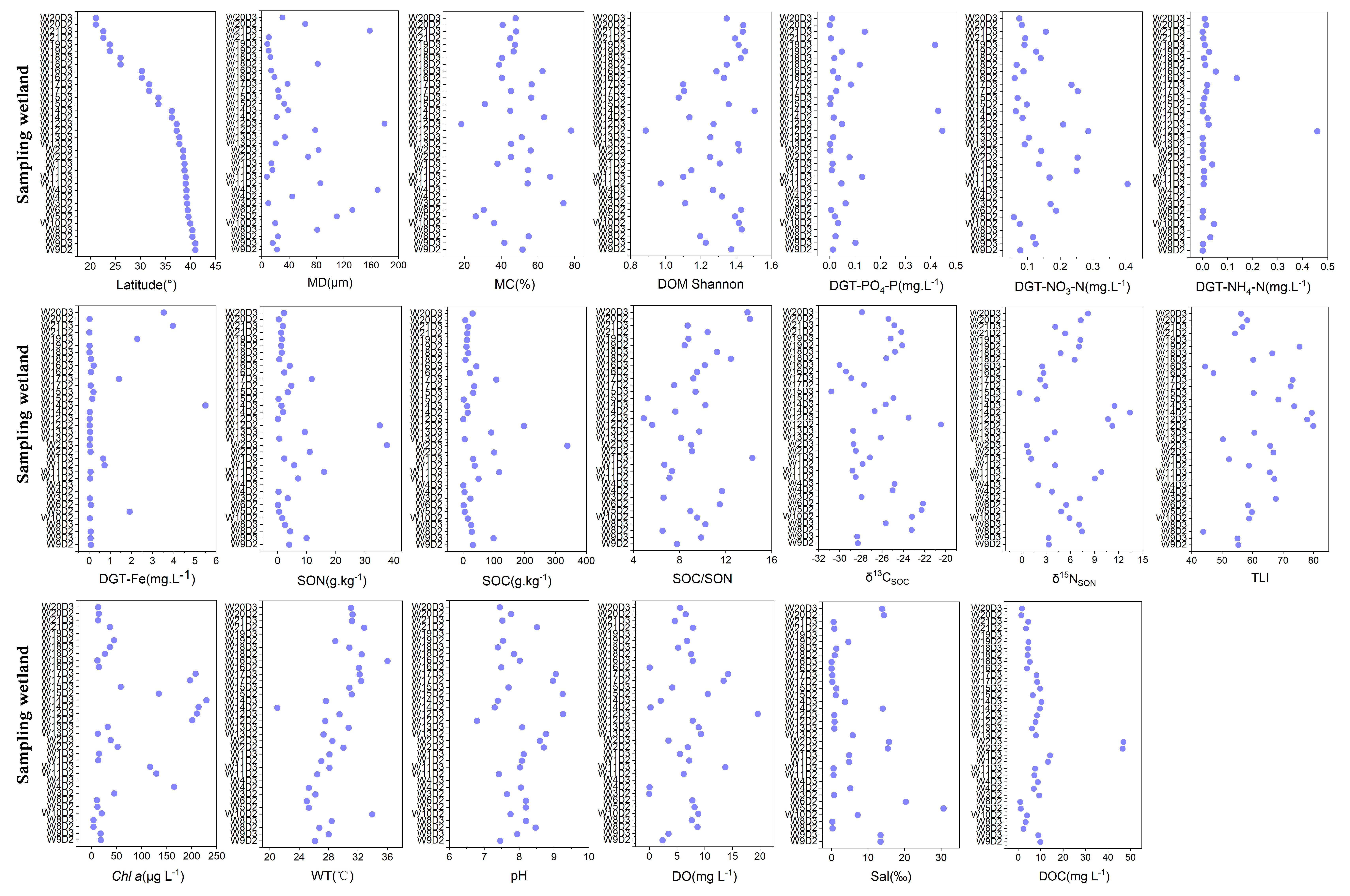


**Fig.S1** Spatial variations in physicochemical parameters and DOM α-diversity index of surface sediments and overlying water in China**^’^**s coastal wetland. The relevant abbreviations are referred to Table 1.


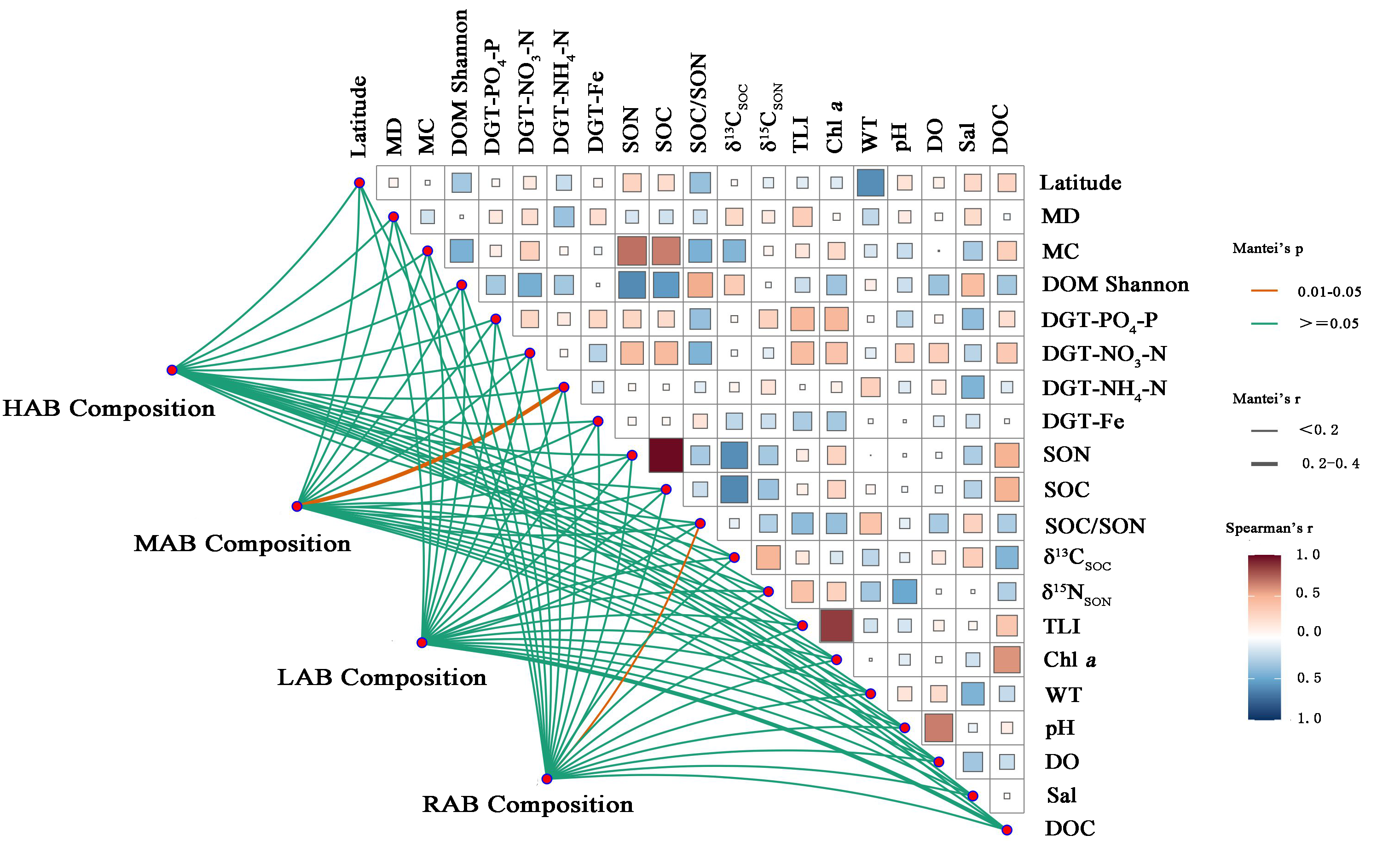


**Fig.S2** Correlation between bacterial community composition and environmental factors. Pairwise comparisons of physicochemical factors are displayed with a color gradient to denote Spearman’s correlation coefficients. Community composition is related to each environmental factor by performing a Mantel test. The relevant abbreviations are referred to the text and Table 1.

**

**

**Fig.S3** The absolute abundance ratio of Archaea/Bacteria+Archaea in China’s coastal wetland water and sediment.





**Fig.S4** Relationships for archaea Shannon diversity similarity and carbon-functional similarity based on Bray-Curtis distance. The regression: y = 0.24 x + 0.06, r^2^ = 0.79, *p* ＜ 0.05.


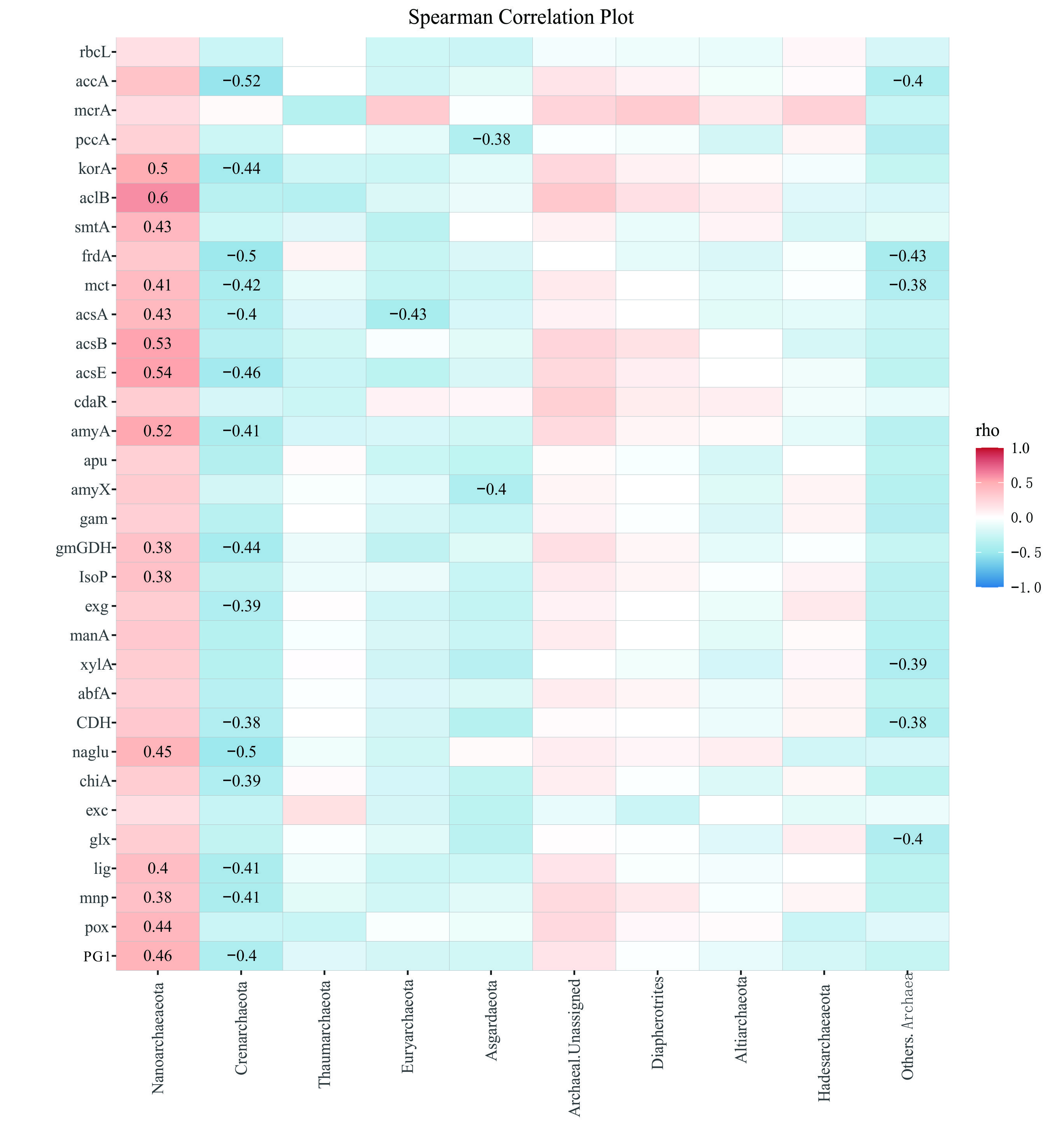


**Fig.S5** Spearman correlation analysis between different archaea phyla and carbon- functional genes with a *p* value ＜ 0.05.





**Fig.S6** Relationship for bacterial community (a), archaeal community (b) and carbon-functional (c) similarity based on Bray-Curtis distance and geographic distance. The regression (bacteria): y = -2.86 × 10^-5^ x + 0.79, r^2^ = 0.02, *p* ＜ 0.05; the regression (archaea): y = -2.2 × 10^-4^ x + 0.82, r^2^ = 0.44, *p* ＜ 0.05; the regression (carbon-function): y = -9.4 × 10^-5^ x + 0.38, r^2^ = 0.05, *p* ＜ 0.05.





**Fig.S7** Relationships for microbial community and carbon-functional similarity based on Bray-Curtis distance. The relevant abbreviations are referred to the text.
